# Personalized and Complex Esthetic Oral Rehabilitation in a Case of Non-Syndromic Oligodontia

**DOI:** 10.3390/jpm14040350

**Published:** 2024-03-27

**Authors:** Silvia Izabella Pop, Ana Procopciuc, Mihai Mițariu, Loredana Mițariu, Radu Vasile Pop

**Affiliations:** 1 Orthodontic Department, Faculty of Dental Medicine, “George Emil Palade” University of Medicine, Pharmacy, Science, and Technology of Targu Mures, 38 Gh. Marinescu Str., 540139 Târgu Mureș, Romania; silvia.pop@umfst.ro (S.I.P.); radupop1982@yahoo.com (R.V.P.); 2 Department of Dental Surgery, Faculty of Dental Medicine, “George Emil Palade” University of Medicine, Pharmacy, Science, and Technology of Targu Mures, 38 Gh. Marinescu Str., 540139 Târgu Mureș, Romania; 3 Department of Dental Medicine and Nursing, Faculty of Dental Medicine, Lucian Balga University, Bd-ul. Victoriei, 550024 Sibiu, Romania; mihai.mitariu@ulbsibiu.ro

**Keywords:** anodontia, oligodontia, oral rehabilitation, dental implants, esthetic dentistry, crowns, ceramics

## Abstract

Dental agenesis is one of the most common developmental anomalies in humans and it is frequently associated with several other oral abnormalities. The present case describes non-familial agenesis of permanent teeth in a twenty-one-year-old boy with no apparent systemic abnormalities. The treatment included a personalized and interdisciplinary approach involving endodontics, orthodontics, implant-supported restorations and prosthetic treatments. The treatment plan was thoroughly elaborated using photographic analysis, study models, orthopantomogram, CBCT and cephalograms. Virtual smile design, diagnostic waxing and mock-ups previsualized the treatment objectives. The edentulous spaces were reconstructed by inserting dental implants and monolithic zirconia implant-supported restorations. The final results showed a highly esthetic and functional rehabilitation. Periodic check-ups have shown that the stability of the result is well maintained and that the implant-supported restorations are an optimal solution for patients with multiple anodontia.

## 1. Introduction

The dental anomaly characterized by the absence of one or more teeth, the total absence of dentition due to the non-formation of dental buds, and the lack of embryonic development or their atrophy, is known in the literature as anodontia [1]. The most common term used to describe missing teeth is anodontia, but terms like oligodontia and hypodontia are also common [2].

Hypodontia is often used as a collective term for congenitally missing teeth, although specifically, it describes the absence of one to six teeth, excluding third molars [1,2,3]. Oligodontia (multiple aplasia) refers to the congenital absence of six or more teeth, excluding third molars. Anodontia represents the complete failure of the development of one or both dentitions [3].

Oligodontia is frequently associated with other oral anomalies, like a reduction in the size and form of teeth and alveolar processes, crowding and/or malposition of other teeth, false diastema, short root anomalies, delayed formation and/or delayed eruption of other teeth, persistent deciduous teeth, anomalies of the enamel, enamel hypoplasia, increased free-way space, deep overbite, taurodontism, maxillary canine/first premolar transposition and altered craniofacial growth [4,5,6].

Underdevelopment of the maxilla in the sagittal, transversal and vertical planes (maxillary hypoplasia) is commonly associated with hypodontia [6]. From a facial aspect point of view, patients with hypodontia exhibit a reduced lower face height, with a hypodivergent vertical skeletal pattern and a class III skeletal anomaly. Upper lip retrocheilia and increased nasolabial angles might be also present [7].

Regarding the treatment objectives, increasing the facial lower third and the posterior rotation of the mandible and decreasing the nasolabial angle are important key points. Addressing these aspects will assure an optimal esthetic and functional result of the treatment of hypodontia.

Space management, which results from the congenital absence of teeth, is often hampered by unfavorable positions of the teeth that are present. In many cases, the orthodontic management of patients with hypodontia can greatly facilitate any restorative treatment [7,8,9,10]. To carry out the treatment of edentulous areas, implant-based prosthodontics are a good option to improve oral function and esthetics in oligodontia [11,12]. Successful implant placement requires both keratinized gingiva and adequate alveolar bone [13]. The remaining deciduous teeth in the alveolar bone have a significant role in maintaining bone width, while their early loss might induce bone resorption [14].

Currently, implant planning software using cone-beam computerized tomography data has made it possible to plan the optimal implant position, taking into consideration the surrounding vital anatomic structures and future prosthetic requirements [13,14].

The main aim of this case report is to highlight the stages of planning and treatment of a 21-year-old patient with hypodontia, involving orthodontics, endodontics, implantology and prosthodontic specialties. The roles of multidisciplinary management and digital planning are highlighted as one of the most important requirements for an individualized treatment of hypodontia.

## 2. Case Presentation

A 21-year-old male patient attended the Natural Smile Dental Clinic, Târgu Mureș, Romania, with an esthetic complaint regarding the congenital absence of several teeth.

This case report is conducted with the informed and signed consent of the patient.

**Figure 1:**
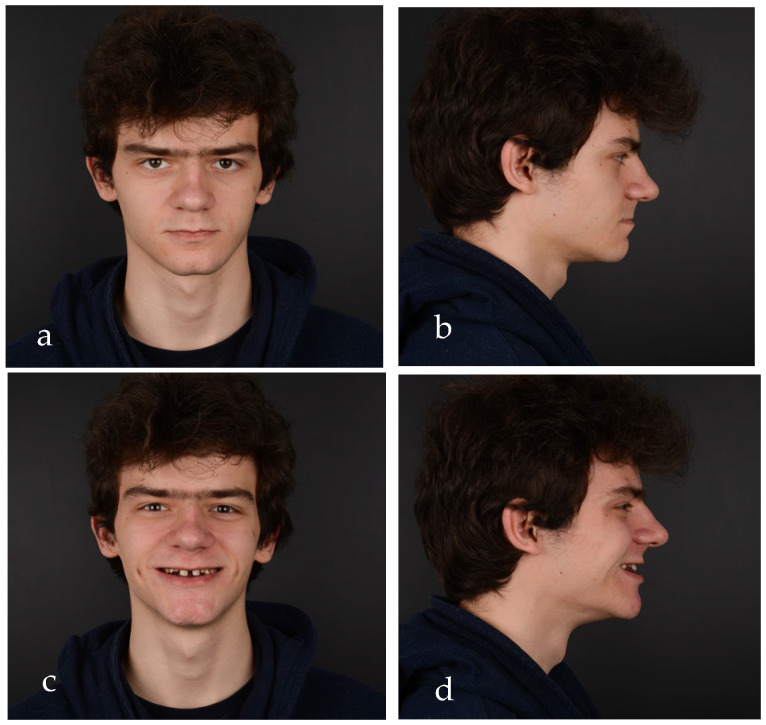
Initial extraoral photos: (**a**) frontal view with lips in contact, (**b**) lateral view with lips in contact, (**c**) frontal view of the smile and (**d**) lateral view of the smile.

**Figure 2:**
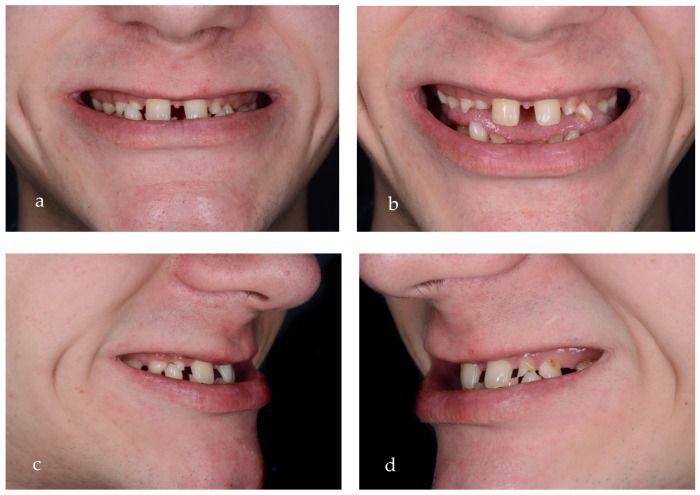
(**a**) Initial smile, frontal view, close-up, highlights the presence of mixed dentition. (**b**) Maximum exposure of the teeth, frontal view, close-up. (**c**) Lateral view of the right side, close-up smile, diastema can be observed. (**d**) Lateral view of the left side, close-up smile, diastema, and caries on temporary teeth can be observed.

**Figure 3:**
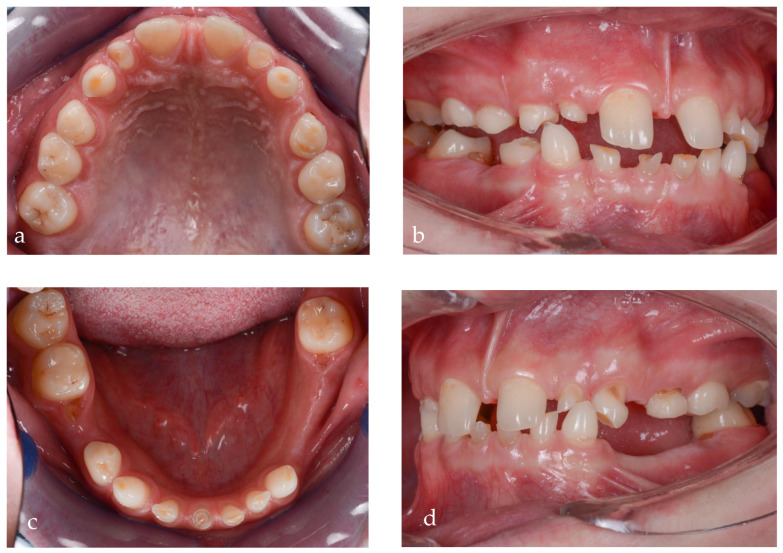
Intraoral pictures of the initial clinical situation: (**a**) upper arch, (**b**) right occlusal view, (**c**) lower arch and (**d**) left lateral view.

**Figure 4:**
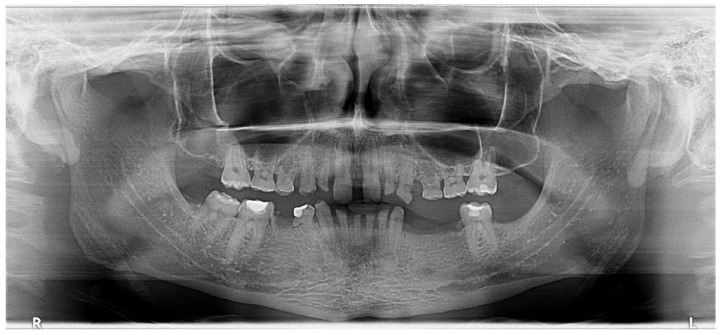
OPG. Radiographic initial situation of the patient. The radiograph reveals the presence of 7 permanent teeth on the dental arches.

**Figure 5:**
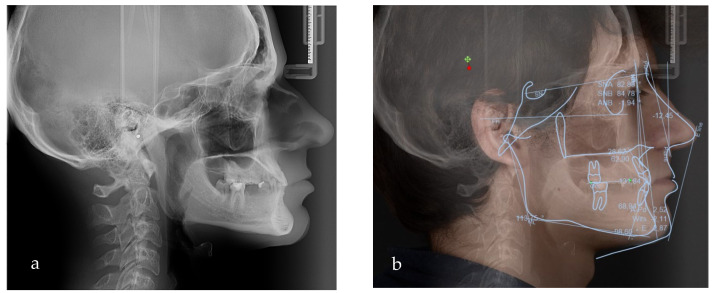
(**a**) Lateral teleradiograph; (**b**) VTO analysis.

After clinical examination, data acquisition was carried out, including taking extraoral (Figure 1a–d and Figure 2a–d) and intraoral photos (Figure 3a–d), OPG (Figure 4), CBCT, lateral teleradiography of the head (Figure 5a) and articulator mounting of study models both in centric relation and maximum intercuspation.

The dental status at the time of the primary consultation was as follows: 16 with occlusal caries, 55 with occlusal caries and abraded occlusal surface, 54, 53 and 52 affected by abrasion, 11 and 21 without pathological changes, 62 and 63 abraded, 64 with occluso-distal caries and abraded occlusal surface, and 26 with old occlusal filling and secondary caries. The missing teeth in the upper arch were 12, 13, 14, 15, 17, 22, 23, 24, 25 and 27. In the lower arch, the following status was present: 36 with old occlusal filling and secondary caries, 73, 72, 71, 81, 82, 83 and 84 affected by abrasion, a residual root from 75, 85 and 46 with occlusal filling and secondary caries, and 4.7 with occlusal caries. The missing lower teeth were 41, 42, 43, 44, 45, 31, 32, 33, 34, 35 and 37 (Figure 3a–d).

From an esthetic standpoint, a low smile line, uneven gingival margins, spaces and incorrect axial inclination of the frontal teeth were present. The position of the Stomion point regarding the incisor position was acceptable.

Radiographic examination did not reveal periapical alteration of the present teeth and excluded the diagnosis of dental impaction (Figure 4).

Based on the clinical and paraclinical examination, the following diagnostics were established: skeletal and dental class III anomaly (ANB angle −1°), with hypodivergent growth pattern and anterior rotation of the mandible (Sn-GoGn 28°), maxillary hypoplasia, slightly protruded upper incisors, diastema, spacing due to anodontia of the lower central incisors, all lateral incisors, all canines, all premolars and three second molars. Therefore, in this clinical case, seven permanent teeth were present.

To evaluate the treatment options and the amount of posterior rotation of the jaws required to improve the facial appearance, the VTO (Romexis) was formed (Figure 5b).

Based on the VTO (visualized treatment objective), protraction and posterior rotation of the maxilla would have been the ideal treatment option in terms of skeletal modifications; however, this treatment option would have needed an orthognathic surgical approach (which was not accepted by the patient).

Therefore, the treatment plan included the following:

1. Treatment of the carious lesions 16, 26, 36, 55, 64, 46 and 47;
2. Preprosthetic orthodontic treatment;
3. Wax-up and mock-up;
4. Endodontic treatment of the lower temporary incisors;
5. Upper and lower implant placement with provisional restorations;
6. Final restoration placement.

**Figure 6:**
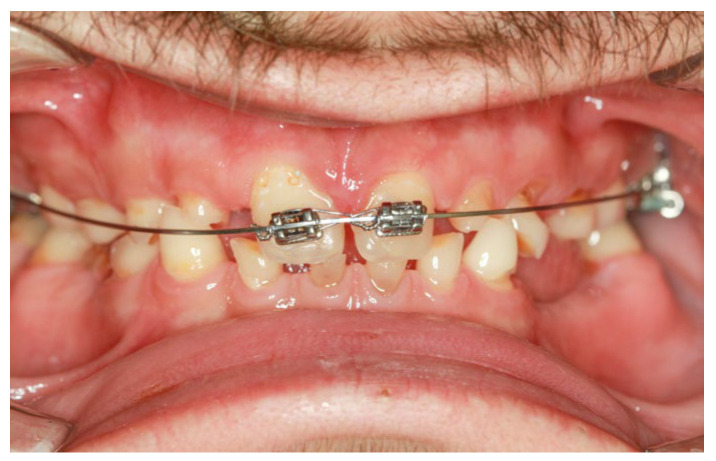
Intraoral clinical aspect during the orthodontic treatment.

Preprosthetic orthodontic treatment was conducted with a partial fixed appliance (transpalatal bar and 0.022-inch Roth prescription brackets on the 11 and 21). The main objective of this treatment phase was to reduce the diastema and slightly retrude the upper incisor to improve the dental position for the final restorations (Figure 6).

**Figure 7:**
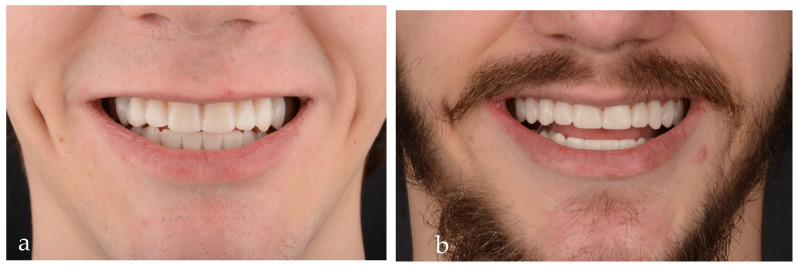
(**a**) Close-up smile with the mock-up. (**b**) Close-up smile with the provisional restorations.

To preview the result and help with the planning of the implant placement but also to establish the new occlusal plane as well as the new vertical occlusal dimension, a diagnostic wax-up was performed after the orthodontic treatment. The wax-up, a fundamental tool in complex oral rehabilitation, was transferred into the patient’s mouth, resulting in the mock-up (Figure 7a), which was also used to make provisional restorations (material used: Luxatemp, DMG Germany) (Figure 7b); this protocol ensures an esthetic and functional result.

**Figure 8:**
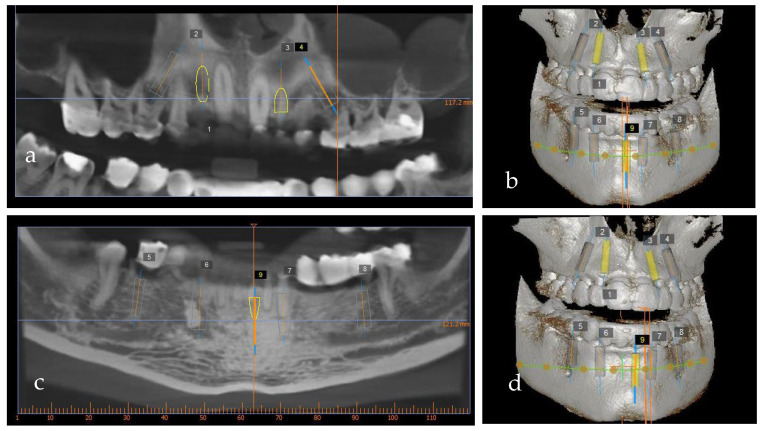
Implant planning software using cone-beam computerized tomography: (**a**,**b**) maxilla and (**c**,**d**) mandible.

**Figure 9:**
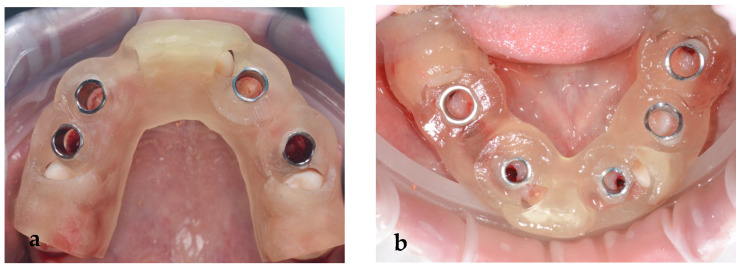
(**a**) Maxillary and (**b**) mandibular surgical guide.

The wax-up also served as a prosthetic guide for the implant position. To precisely plan the implants’ placement from a prosthetic point of view, a CBCT (PaxFlex 3D, Vatech, Hwaseong-si, Republic of Korea) was performed and an initial digital planning software analysis was conducted with Easy3DPlus version 1.2 (Vatech, Hwaseong-si, Republic of Korea) (Figure 8a–d). Based on the previsualized implant placement, surgical guides were designed (Figure 9a,b), to achieve exact implant placement. The guides were designed in the Blue Sky Plan^®^ software version 4.9.4 (Libertyville, IL, USA) and the surgical templates were fabricated using Dental SG resin (Formlabs, Somerville, MA,USA).

Using digital planning, the placement of nine implants, four on the maxilla and five on the mandible, was established (Figure 8a–d) based on the position of the provisional restorations. The implants were planned to be placed in the optimal prosthodontic position; tooth size, bone quality and volume, and the location of the mandibular nerve and sinus were also taken into consideration.

Two separate surgical procedures were planned. The first implant insertion surgery was performed for the upper jaw; four MIS V3 (MIS Implants Technologies UK Ltd., London, UK, implants were inserted in the position of 12, 14, 23 and 24, with two MIS Connect abutments (MIS Implants Technologies Ltd., London, UK) on 12 and 13 and two angulated multiunits on 14 and 24. To restore the patient’s esthetics, a temporary PMMA (Dentsply Sirona, Charlotte, NC, USA) bridge was used for the upper arch. After the implant placement, a period of 6 months of healing was decided.

Decision making regarding the temporary teeth was a difficult process. Compromised teeth due to abrasions or caries, as well as those positioned in the strategic positions of dental implant placement, were extracted. Only 55 and 65 were preserved to be prosthetically restored with dental crowns because of their well-preserved roots.

Several PET (Partial Extraction Therapies) techniques [15] for the temporary teeth were used. The socket shield technique was chosen for the teeth 52 and 63. This technique pioneered by Hürzeler [16] demonstrated that retaining the buccal aspect of the root during implant placement does not appear to interfere with osseointegration and may be beneficial in preserving the buccal bone plate [16].

**Figure 10:**
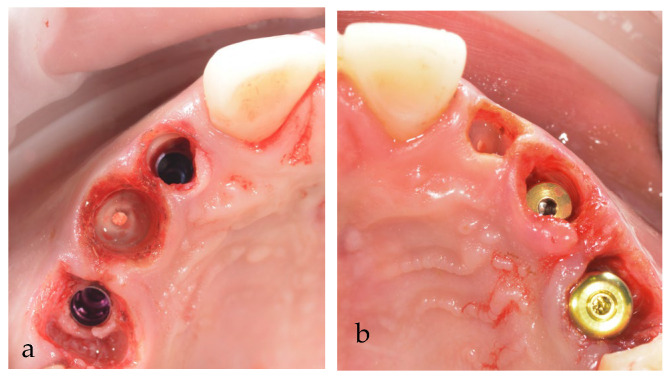
Maxillary submerged roots (PET) of temporary teeth: (**a**) first quadrant; (**b**) second quadrant.

As the alveolar ridge resorption is an unavoidable consequence of tooth extraction, we also decided to perform another PET type of surgery, root submergence of the 53 and 62 temporary teeth, as a technique for ridge preservation [15]. The teeth were endodontically treated before the surgery (Figure 10a,b).

**Figure 11:**
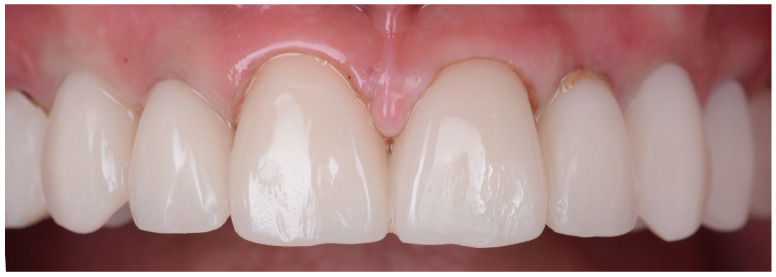
PMMA immediate provisional restoration.

Because sufficient primary stability was not achieved, only less than 30 Ncm, the patient received a temporary PMMA bridge splinted on all of the remaining teeth (Figure 11) on the second day after the surgery, after an analog impression. This was an important aspect for functional and esthetic reasons to preserve the soft tissues but not to exert any pressure on the implants.

For the mandibular arch, five MIS V3 implants were inserted with guided surgery in the position of 36, 34, 32, 43 and 45, all with 40 Ncm primary stability. We used three MU abutments and two Connect abutments for a passive fit of the future screw-retained bridge and also to have the benefits of the one abutment one time concept.

**Figure 12:**
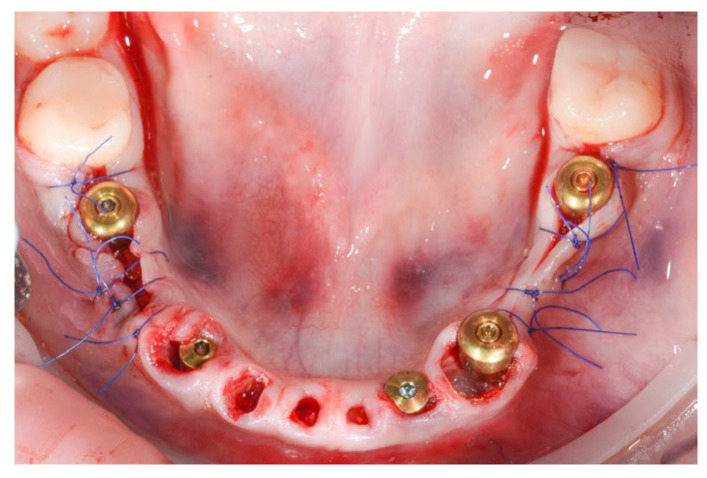
Post-operative aspect of mandibular implant placement and PET for 71, 72, 73, 81, 82 and 83.

The PET technique was used for the remaining mandibular incisors and canines after previous endodontic treatment (Figure 12).

**Figure 13:**
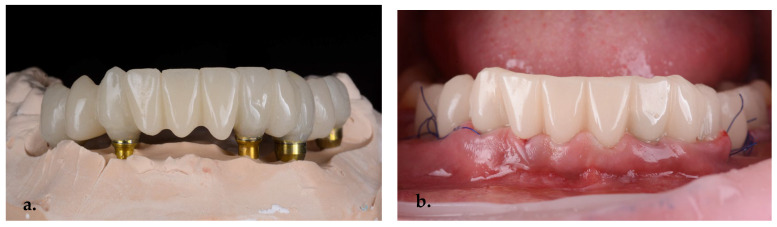
PMMA screw-retained second-day provisional bridge (**a**) on the stone model and (**b**) an intraoral view.

On the second day, an immediate PMMA screw-retained provisional restoration was inserted to preserve the gingival margins and to guide the emergence profiles, as well as to ensure the patient’s esthetics and function during the healing period (Figure 13a,b).

**Figure 14:**
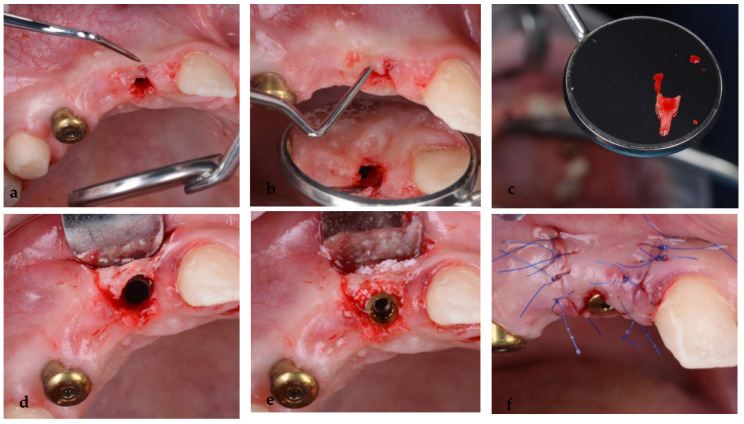
(**a**,**b**) Infection at the level of the vestibular shield, (**c**) extraction of the vestibular portion of the root 52, (**d**) curettage, (**e**) bone grafting and (**f**) sutures.

Six months after the placement of the maxillary implants, at the uncovering stage, a complication occurred at the level of the implant placed in the position of tooth 12, where a socket shield was performed. Clinically, a fistula appeared at the level of the vestibular mucosa, where the buccal portion of the root was retained. A minimal lap was made, followed by the extraction of the root, curettage, bone grafting and passive sutures. The stability of the implant was not affected (Figure 14a–f).

**Figure 15:**
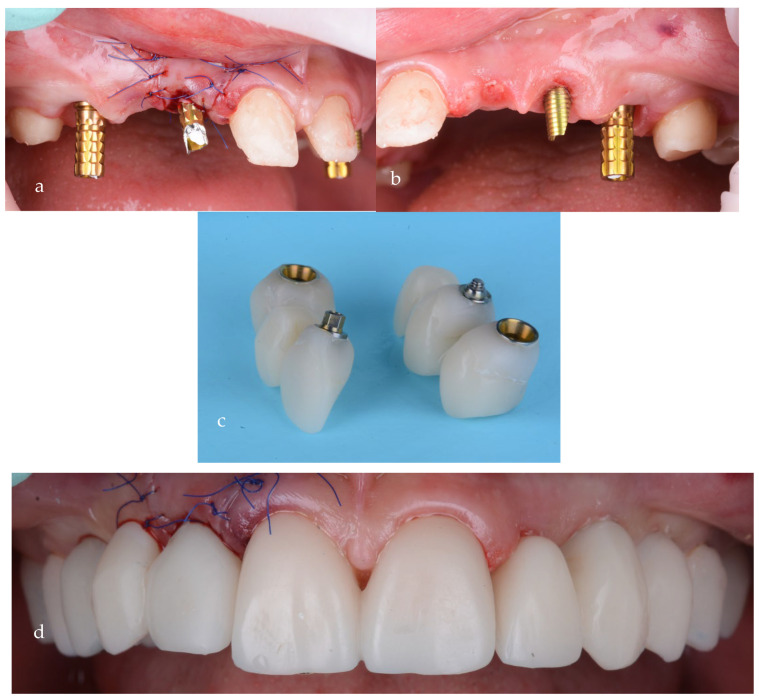
Screw-retained provisional bridges on temporary abutments: (**a**) first quadrant, (**b**) second quadrant, (**c**) provisionals and (**d**) after intraoral cementation.

Temporary screw-retained bridges, with proper emergence profiles, were used at this stage for soft tissue shaping of the gingival contour (Figure 15a–d).

**Figure 16:**
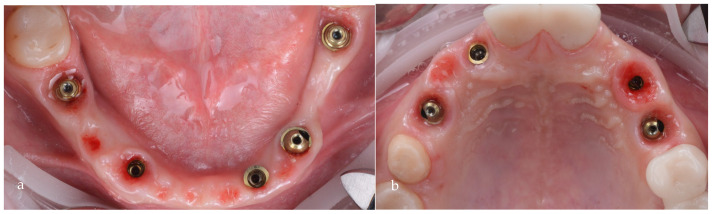
Healed emergence profiles: (**a**) mandible; (**b**) maxilla.

**Figure 17:**
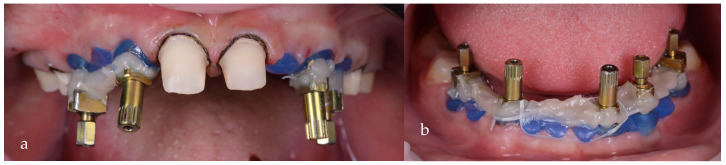
Analog impression with direct transfer of the emergence profile: (**a**) maxilla; (**b**) mandible.

After 3 more months of healing, the proper shaping of the tissue could be observed (Figure 16a,b). At this stage, an impression for the prosthetic restorations was taken (Figure 17a,b).

**Figure 18:**
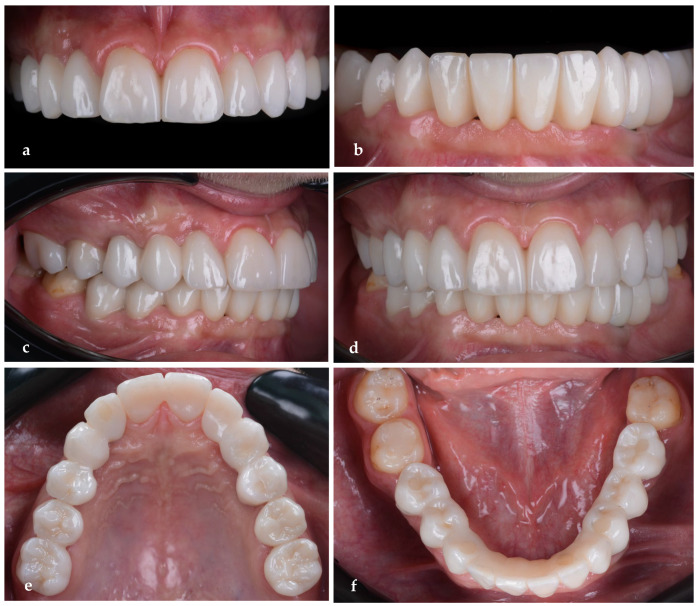
Intraoral pictures of the result of monolithic zirconia-fixed partial dentures on teeth and implants: (**a**) upper incisors, (**b**) lower incisors, (**c**) right lateral view, (**d**) frontal occlusal view, (**e**) upper arch and (**f**) lower arch.

For the final restorations, monolithic zirconia was used for optimal biologic compatibility and superior strength (Figure 18a–f). The patient’s esthetics and function were fully restored. The intraoral pictures showed significant improvement in the micro esthetical characteristics. The gingival margins were levelled, and the teeth axes were corrected.

The total treatment time was 26 months, of which 3 months included the preorthodontic treatments, 7 months comprised the orthodontic treatment, and 16 months was the duration from the initial implant placements to the final restorations.

**Figure 19:**
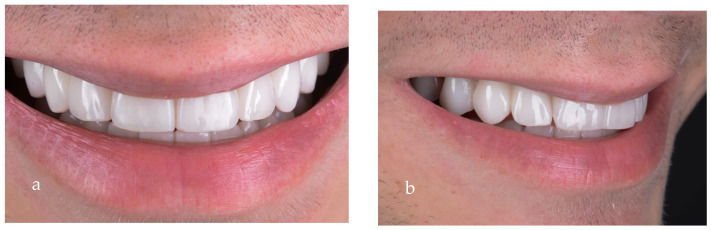
Close-up of the final smile: (**a**) frontal view; (**b**) lateral view.

**Figure 20:**
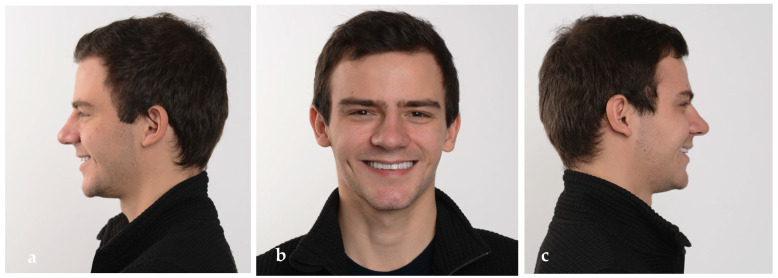
Extraoral pictures of the result: (**a**) left lateral view, (**b**) frontal view and (**c**) right lateral view.

The extraoral pictures of the smile (Figure 19a,b) and of the face (Figure 20a–c) showed significant improvement in the dentolabial esthetics. The facial lower third was optimized due to the increased occlusal vertical dimension and slight posterior rotation of the mandible. The smile line became convex and parallel with the lower lip, while the profile became straight. The correct dental axes of the upper incisors improved the lip support.

**Figure 21:**
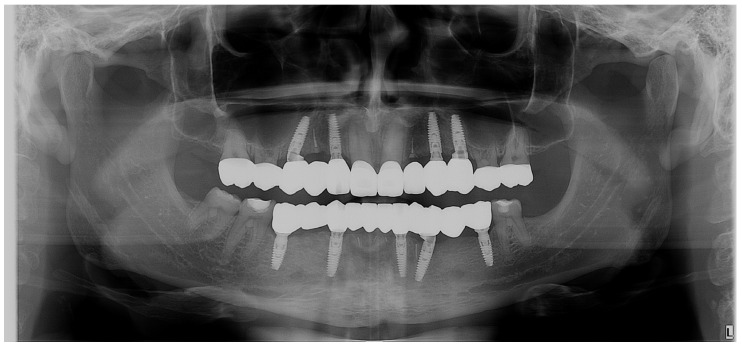
Control OPG: the final situation after the implant–prosthetic treatment.

The radiological evaluation (Figure 21) of the final restorations showed good osseointegration of the implants with optimal bony peaks. Also, the submerged roots can be observed for the PET used to maintain the tissues in place.

**Figure 22:**
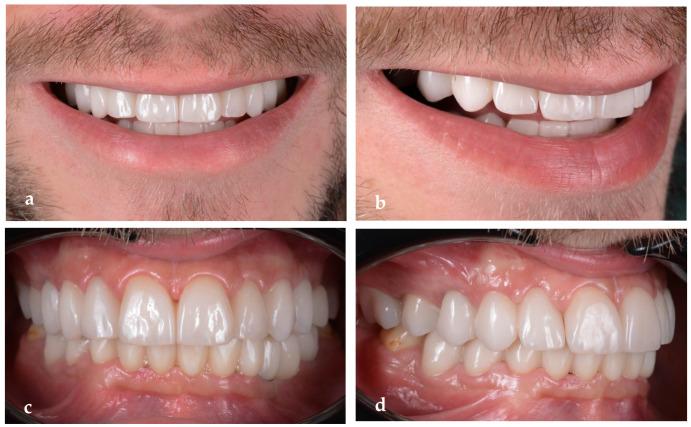
(**a**,**b**) Smile and intraoral (**c**,**d**) images at the one-year follow-up.

The one-year follow-up (Figure 22a–d) showed a good stability of the rehabilitation from an esthetic, functional and biologic point of view. The patient’s quality of life was well improved, and his self-esteem grew as he became a confident adult.

**Figure 23:**
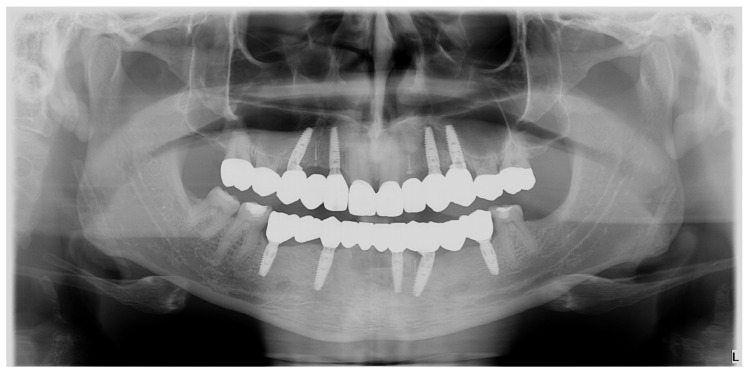
OPG: three-year radiological recall.

**Figure 24:**
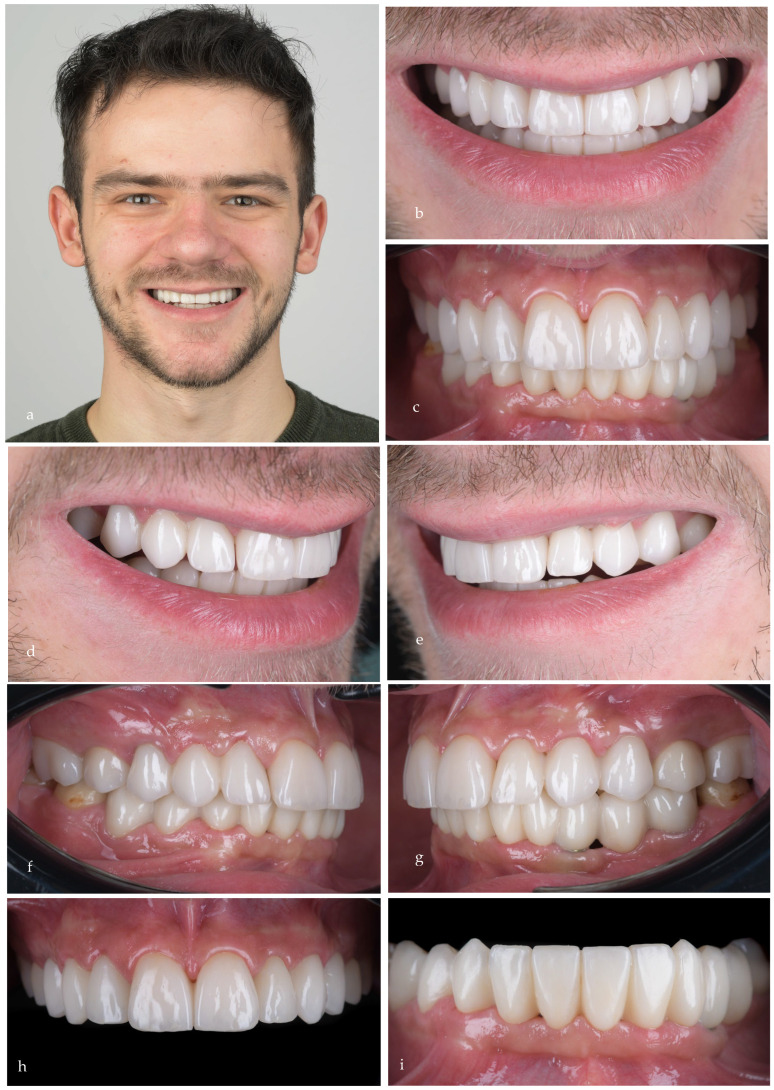
Images at the three-year follow-up: (**a**) extraoral frontal view of the smile; (**b**)close-up frontal view of the smile, (**c**) frontal occlusal view; (**d**) right lateral view of the smile; (**e**) left lateral view of the smile; (**f**) right occlusal view; (**g**) left occlusal view; (**h**) upper incisors; (**i**) lower incisors.

Also, at the three-year recall, the radiological (Figure 23) and clinical (Figure 24) result was well maintained.

## 3. Discussion

Hypodontia has a great impact on the quality of life of the patients, affecting both their physical and emotional well-being. From a functional point of view, occlusion and speech are affected [17,18,19]. The extraoral and intraoral esthetic modifications may cause emotional problems such as low self-esteem and altered behavioral patterns [20,21,22]. Therefore, functional and esthetic rehabilitations are essential for a successful treatment outcome.

Patient motivation is also an important factor in obtaining a good treatment outcome. Complex treatment of hypodontia requires longer periods and numerous appointments [21]. Maintaining oral hygiene is also a key point for successful treatment outcomes. The patients’ concerns and expectations regarding the treatment need to be taken into account when treatment plans are discussed [19].

The placement of implants in edentulous patients must be very precise to eliminate lesions of nearly located structures (vessels and nerves) [23,24,25]. Therefore, pretreatment planning is as important as the surgical part of the insertion [26]. The tools providing exact three-dimensional information for the optimal prosthetic-driven placement of implants are recommended to be used [27]. The use of computed tomography (CT), cone-beam computed tomography (CBCT) and 3D implant planning software significantly reduces the complications of these surgical procedures [28].

There are several advantages of guided implant placement such as increased precision, reduced trauma to the patient and reduced duration of the surgical procedure [27]. However, the higher cost of this approach might be considered a disadvantage [27]. In the case of our patient, a guided surgical approach facilitated the precise placement of the implants, especially from a prosthetic point of view.

A multidisciplinary approach has several advantages when severe hypodontia cases are treated. In the present case, several specialties contributed significantly to obtaining a final esthetic and functional result. The endodontic treatment of the remaining primary teeth allowed for good alveolar bone preservation. The orthodontic treatment, although limited to the upper arch, allowed for the repositing of the upper incisor and reduced the diastema. Without repositioning, the upper incisor micro esthetical characteristics, in terms of tooth width/height ratio would have remained altered.

A visual plan can be a helpful when interdisciplinary cases are treated, especially in predicting the goals that need to be achieved for the patient. The use of the VTO in the present case allowed the dental team to visualize the amount of mandibular clockwise rotation due to the increase in the occlusal vertical dimension. It also allowed them to establish alternative treatment options, such as surgical maxillary advancement and maxillary clockwise rotation, a treatment option rejected by the patient.

The socket shield technique used in this case was described by Hürzeler [16]. This treatment option was based on fact that the root submergence is indicated for preservation of the alveolar ridge beneath full dentures and fixed or removable partial dentures [29,30]. Hürzeler’s [16] study showed several advantages of this technique: the formation of a bony layer between the socket shield and the implant surface and a reduction in the need for invasive bone grafts around implants, especially in the esthetic zone. Any active infection of the root and the apical area must first be resolved by endodontic treatment. An adequately root-treated tooth or a vital, infection-free tooth root may be submerged [29].

Stability of the case was shown in the follow-ups, both at 1 and 3 years post-treatment. Patient satisfaction was high, and the quality of his life increased significantly.

## 4. Conclusions

This case presentation shows a complex interdisciplinary approach of severe hypodontia, where the digital tools (VTO, CBCT and implant planning software) used for treatment planning are represented.

## Data Availability

The data presented in this study are available on request from the corresponding author due to patient’s privacy.
